# Gaming time and impulsivity as independent yet complementary predictors of gaming disorder risk

**DOI:** 10.1038/s41598-024-81359-1

**Published:** 2025-01-22

**Authors:** Paulina Daria Szyszka, Aleksandra Zajas, Jolanta Starosta, Patrycja Kiszka, Sylwia Starzec, Paweł Strojny

**Affiliations:** 1 Institute of Psychology, Faculty of Philosophy, Jagiellonian University, Kraków, Poland; 2 Institute of Applied Psychology, Faculty of Management and Social Communication, Jagiellonian University, Kraków, Poland; 3 Doctoral School in the Social Sciences, Jagiellonian University, Kraków, Poland

**Keywords:** Psychology, Human behaviour

## Abstract

Prolonged gaming time, along with increased impulsivity—a key element of poor self-regulation—has been identified as linked to gaming disorder. Despite existing studies in this field, the relationship between impulsivity and gaming time remains poorly understood. The present study explored the connections between impulsivity, measured both by self-report and behavioral assessments, gaming time and gaming disorder within a cohort of 82 participants. While gaming time exhibited a significant correlation with gaming disorder, only self-reported measures of impulsivity and one behavioral metric showed a correlation with gaming disorder. Self-report measures of impulsivity exclusively predicted gaming disorder when included in a regression model with gaming time. The interaction between gaming time and impulsivity, aside from one behavioral metric was deemed insignificant. These findings suggest that impulsivity and gaming time, although associated with gaming disorder risk, are independent variables. Further research should aim to clarify these relationships and explore potential interventions targeting both DGI and impulsivity to mitigate gaming disorder risk.

## Introduction

The realm of gaming has emerged as an increasingly favored pastime, with the global community of gamers surpassing a staggering 3 billion in 2023, a figure anticipated to soar beyond 3.3 billion by the close of 2024^1^. As this phenomenon unfolds, researchers delve into the intricate interplay between gaming and various factors, such as cognitive functioning. Initially, scholarly attention gravitated towards the prospect of gaming fostering cognitive skills^2,3^. While some studies have highlighted the positive impacts of gaming on cognitive functioning^4–7^, recent research has underlined the necessity for caution in hailing gaming as a universal solution for skill development^4^. It is apparent that gaming and certain cognitive abilities are interconnected, but further exploration and development of theoretical frameworks are required to construct a comprehensive explanatory model^5^. Moreover, this interconnection may be explored from other perspectives, including the possible relationship between cognitive characteristics and susceptibility to disordered gaming.

### Gaming disorder

As researchers delve into the intricate relationship between gaming and various cognitive factors, one aspect stands out—the addictive nature of gaming. Gaming disorder (GD) is a condition recognized by international health classification systems, with the World Health Organization officially acknowledging it in the 11th International Classification of Diseases (ICD-11) in 2022^6^. In essence, it is characterized by patterns of behavior that mirror addictive disorders, involving the following symptoms: (1) difficulties in controlling gaming behavior (such as its onset, frequency, intensity, etc.); (2) giving more importance to gaming to the extent that it supersedes other interests and everyday activities; (3) continuing or intensifying gaming despite the occurrence of negative outcomes. Accordingly, in line with other addictive behaviors, the transition from controlled gaming to addiction is multi-faceted and raises a fundamental question: “What factors contribute to this phenomenon?“.

Therefore, one perspective on the development of gaming disorder involves pinpointing the moments when self-regulation begins to fail. A promising avenue of investigation dedicated to addressing this question delves into the examination of low cognitive skills as potential contributors to the development of GD, with initial findings supporting this hypothesis^7^. Within the spectrum of gaming disorders, a multitude of challenges arises, prominently encompassing difficulties related to self-control across various levels. These challenges range from struggling to resist persistent thoughts about gaming to grappling with the inability to adhere to self-imposed gaming limits^8^. Given these complexities, the concept of self-regulation emerges as a central focus for researchers, further enriching our understanding of the complex dynamics of problematic gaming.

#### Self-regulation as an explanatory factor for GD

One of the reasons for the transition from non-problematic video game play to GD is malfunctioning self-regulation mechanisms^9^. Considering the cognitive perspective of behavioral addictions (including GD), self-regulation is recognized as playing a pivotal role^10^. The reason is that self-regulation is responsible for the balance of the reflexive-controlled and impulsive-automatic systems^11^. Recent research into the specific neurocognitive processes involved in addictive disorders has challenged the traditional dual-process framework, suggesting its incompleteness^10–12^. Instead, a tripartite model has been proposed, integrating a third interoceptive system. As illustrated, self-regulation is a multifaceted mechanism, even considered an umbrella construct encompassing both automatic and controlled processes^13^. This complexity is particularly evident in problematic gaming, where various aspects of self-regulation come into play. These include high sensation-seeking^13^, low impulse control^14^, emotional dysregulation^15–18^, and low self-control^7,19–22^.

### Associations between GD and impulsivity

As evident from the arguments above, various facets of self-regulation can be distinguished. Among these, impulsivity is characterized as a key component of poor self-regulation^23,24^. Given our focus on this aspect in the reported study, we delve into its association with GD in the subsequent section.

There is a substantial body of empirical evidence confirming the relationship between impulsivity and GD. A recent review^25^ found that of 33 published studies, only one failed to confirm this association^26^. The remaining studies consistently reported some form of relationship between impulsivity and GD. Moreover, seven studies identified impulsivity as a predictor of GD^27–33^. In more complex models, the association between impulsivity and GD was found to be mediated by self-regulation^31^ or interpersonal relationships and depression^33^. In one instance, impulsivity moderated the link between affect and GD^29^. It is noteworthy that operationalizations of impulsivity vary across studies, often divided by the method of data collection; while some researchers employ objective methods like computer tasks, others rely on self-report measures. According to the review results, self-report tools are much more prevalent, used in 29 out of 33 analyzed studies, while computer tasks were utilized in 11 cases^25^.

Since impulsivity may be expressed as succumbing to impulses^34^ and gaming activity control is related to the management of impulses, it could be expected that these gamers who are less efficient in impulse control may face stronger consequences of gaming, including the heightened risk of developing GD. If two players with similar levels of gaming involvement (understood as time spent playing) exhibit different levels of impulsivity, it can be assumed that the same time spent gaming, in a controlled or impulsive manner, may result in more severe consequences for the latter. These consequences should be revealed during GD questionnaire assessments. It is also in line with the I-PACE model, which posits general inhibitory control as a moderator of the relationship between the stimulus triggering the desire to play and the psychological effects of gaming^9^. This rationale underpins our expectation that impulsivity moderates the relationship between gaming involvement and GD risk.

### Gaming involvement

Gaming involvement typically refers to the amount of time spent gaming per day or per week. This direct gaming involvement (DGI) has been identified as one of the most significant variables associated with Gaming Disorder^36,37^. Consequently, individuals who spend more time gaming tend to exhibit more severe symptoms of GD^20,38^. However, Pontes et al.^39^ and Strojny et al.^40,41^ indicate that gaming time alone is not a sufficient predictor of Gaming Disorder. In line with the I-PACE model^9^, it is essential to consider other variables such as psychopathology, coping mechanisms, temperamental factors, or specific needs, motives, and values when seeking predictors of GD. Recent research shows that depression, anxiety, ADHD, loneliness, interpersonal conflicts may serve as risk factors for GD^42–45^. Furthermore, the motivation to escape has been identified as one of the most significant factors in behavioral addiction^46^. Moreover, the ability to fulfil personal needs within a game and develop self-esteem based on achievements in-game may also contribute as risk factors for GD^42,46^.

Behaviors only indirectly related to gaming (IGI - indirect gaming involvement^40^), such as watching streams on platforms like Twitch or Youtube, thinking about games, talking about games on social medias or forums, reading about game lore, strategies or theories, or considering purchasing game-related merchandise or collectibles (e.g. skins) may also hold predictive value for developing GD. Such behaviors could occur especially when individuals are unable to engage in direct gaming activities. It could be related to such symptoms of addiction as preoccupation and loss of other interests^6,47^. In such cases, gaming activities—both direct and indirect—become the center of the individual’s attention^48^.

### Gaming involvement and impulsivity

Impulsivity stands out as a major risk factor for Gaming Disorder^25^. The frequency of gaming has been identified as a partial mediator in the relationship between impulsivity and GD in the research conducted by Blinka et al.^27^. Müller et al.^49^ indicate that gamers characterized by high levels of general motor impulsivity tend to experience difficulties in inhibiting behavioral responses when confronted with stimuli related to gaming, potentially including behaviors indirectly related to gaming involvement (IGI). Furthermore, Li et al.^50^ found impulsivity to be associated with both gaming time and GD, with GD also linked to DGI. Furthermore, Kräplin et al.^51^ indicate that lower inhibitory control predicts greater time spent gaming. On the other hand, Raybould et al.^52^ demonstrated that positive urgency, understood as the tendency to engage in impulsive actions in response to positive emotions, and delay discounting, are associated with higher levels of direct gaming involvement (DGI).

### Present study

Similarly to other cognitive characteristics of gamers, impulsiveness itself cannot be considered either necessary or sufficient for the development of GD. The primary determinant in this process lies in the direct engagement with gaming. In simpler terms, one cannot become addicted to something with which they have had no interaction. Consequently, isolating impulsiveness as a predictive factor for GD, independent of gaming time, lacks validity. Nevertheless, impulsivity may be associated with the development of GD^25^. We aimed to take closely examine this potential relationship, particularly by capturing different facets of impulsivity in our measurements. This includes results from classic computer tasks, which serve as objective indicators of impulsivity, as well as self-report measures, which are less susceptible to distortion due to participants’ computer proficiency.

Our initial objective was to examine simple relationships between various aspects of impulsivity and GD risk. However, in line with the interactional-transformational model, which underscores the pivotal role of multifaceted factors in the genesis of pathological behaviors^53^, we anticipated that they function as moderators, influencing the strength and/or direction of the relationship between gaming involvement and the risk of GD^54^. This mirrors the role of other moderating factors in the interplay between direct gaming involvement and GD risk^7,41,55^. To date, no studies have reported findings that investigate the relationship we propose.

In summary, we predicted that both DGI (Hypothesis 1) and impulsiveness (Hypothesis 2) would correlate with GD risk. Moreover, adding impulsiveness-related variables along with DGI to the regression model explaining GD risk would improve its fit (Hypothesis 3). Finally, impulsivity will act as a moderator of the relationship between DGI and GD risk (Hypothesis 4).

Additionally, due to the fact that the presented study represents a novel exploration in this direction, we also sought to explore the relationships between GD risk and other characteristics, including self-control, emotional regulation, behavioral inhibition/activation, and deficits in executive functioning.

## Methods

### Participants

Participants were recruited among Jagiellonian University students via electronic communication and were offered a net compensation of 35 PLN. Subjects had to be of legal age to take part in the study. Their age was verified by document checks during compensation payments.

A sample size of *N* = 73 was calculated for regression and moderation analyses using G*Power^57^. Results indicated the required sample size to achieve 80% power to detect a medium effect size, at a significance criterion of α = 0.05.

Our final sample included 82 individuals aged (23 women, 56 men, 2 individuals identifying with another gender, and 1 participant who refused to disclose their gender) between 18 and 36 consisting of both working (56.1%) and non-working (43.9%) students. More than half were in a relationship (57.3%).

### Data exclusion criteria

According to the preregistration data exclusion criteria for computerized tasks (Stop-Signal, Go/No-Go, Delay and Probability Discounting) were as follows:

(1) Failure to complete the procedure, responding consistently, or displaying outlier results (3rd SD), in particular:

In the Stop Signal Task, instances when the average RT on unsuccessful stop trials exceeded the average RT on go trials and/or the participant omitted more than 20% “go” trials. There were two such cases and six instances of missing SST data, two of which were attributed to technical issues in the participant’s browser.

In the Go/No-Go task, cases where the participant omitted more than 20% “go” trials. Two such cases were identified along with one case of missing data.

(2) At least one failed attention check. There was one participant who failed the attention check.

### Procedure

The study was carried out online. Each participant was individually connected to the experimenter via their preferred voice connection method, such as phone, Discord, or Google Meets. The experimenter remained connected throughout the procedure.

Several measures were implemented to ensure data quality. Participants were informed that experimenters could verify data entries at any time and were encouraged to report any issues during the procedure. Prior to the study, each participant received a checklist outlining key conditions: the procedure must be conducted on a computer placed on a table (not on a sofa or similar surface); Google Chrome or Firefox browsers must be used with all tabs closed except for the one containing the study procedure; the surroundings must be quiet, and participants should be in a suitable condition; smartphones should be silenced and out of sight.

After consenting to the study, participants provided their age and gender. They then completed three computerized tasks in the following order: Stop-Signal Task, Discounting Task (data from the task turned out to be unusable due to a technical error during its programming, hence omitted from further analysis), and Go/No-Go Task. Subsequently, participants were automatically directed to Qualtrics platform to complete questionnaires in the following order: Gaming Disorder Test^57^, Gaming Involvement Scale^40^, Peripheral criteria of GD^58^, Barkley Deficits in Executive Functioning Scale – Short Form (BDEFS-SF)^59^, NAS-50^60^, Emotion Regulation Questionnaire (ERQ)^61^, Barratt Impulsivity Scale - Brief (BIS-Brief)^62^, Behavioral Inhibition/Activation System (BIS/BAS)^63^, Self-Control Scale Short Form (SCS-SF)^64^, Adult ADHD Self-Report Scale (ASRS-v.1.1)^65^ and Patient Health Questionnaire (PHQ-4)^66^. Finally, participants provided information about their preferred gaming platform, preferred genres, and demographic details. Participants were compensated after completing all questionnaires.

### Measures

Due to the fact that this study is the first in a series, we decided to use several different measures of impulsivity. Details of all measurements used in the study are provided in Table 1 (hypotheses testing) and Table 2 (exploratory analysis).

**Table 1:** Variables representing impulsivity in our study.

Measurement tool	Outcome variables
Stop signal task	average time to stop a response, i.e. stop-signal reaction time (Stop Signal Reaction Time; SSRT)
	proportion of incorrect responses on go trials with a response (SST Go Error)
Go/No-Go	mean reaction time (GNG Go RT)
	Proportion of correct responses on Go trials (GNG Go Accuracy)
	Proportion of successfully inhibited responses (GNG Stop accuracy)
NAS-50 (one subscale)	Inhibition and Adjournment
BIS-Brief	Impulsivity in action
	Self-control

**Table 2:** Variables used for exploratory purposes.

Measurement tool	Outcome variables
Stop signal task	Average delay between the presentation of the go stimulus and the stop-signal in ms (Stop Signal Delay; SSD)
	Reaction time on go trials with a correct response in ms (SST Go RT)
Go/No-Go	Total Accuracy (GNG Total Accuracy)
Peripheral criteria of gaming disorder	Peripheral GD
BDEFS	BDEFS Self Management Time
	BDEFS Organization ProblemSolving
	BDEFS Self Restraint
	BDEFS Self Motivation
	BDEFS Self Regulation Emotions
NAS-50	Proactive Control
	Goal Maintenance
	Initiative and Persistence
	Switching and Flexibility
	Self-control General
ERQ	Cognitive Reappraisal
	Expressive Suppression
	Emotional Regulation General
BIS-BAS	Behavioral Activation Drive
	Behavioral Activation Fun Seeking
	Behavioral Activation Reward Responsiveness
	Behavioral Inhibition
SCS-SF	Self-control
Adult ADHD Self-Report Scale	ADHD
PHQ-4	Depression and Anxiety

#### Computerized tasks

Two computerized tasks were used: the Stop-Signal Task (SST)^67^ and the Go/No-Go Task (GNGT)^68^. Both tasks operated in full-screen mode and lasted approximately 20 min each, with a maximum 5-min break between tasks. The tasks were conducted on secure servers specifically designed for online studies: SST on Jatos (written in jPsych by Verbruggen et al.^69^; “STOP-IT” implementation) and the GNG on Pavlovia (written in PsychoJS).

##### Go/no-go task

The Go/No-Go task was based on the version presented by Li et al.^70^. The task included one training block of 20 trials and two experimental blocks of 90 trials each (in sum, 200 trials). After each block, a pause was introduced, with a duration chosen by the participant. In the instructions, it was emphasized that participants should try to be as fast and accurate as possible. Reaction time shorter than 100 ms in “go” trials were omitted from analysis.

Each trial began with a fixation cross shown for 700 ms, followed by a triangle pointed up (“go” trial) or down (“no-go” trial) shown for 200 ms, and another fixation cross shown for 1700 ms. Participants were instructed to press the spacebar on “go” trials while refraining from responding on “no-go” trials. The proportion of “go” and “no-go” trials was equal, with the restriction of a maximum of three consecutive repetitions of the same trial type.

##### STOP-signal task

The “STOP-IT” jPsych implementation of the SST was used^70^. The task comprised one practice block of 32 trials and four experimental blocks of 64 trials each (in sum, 256 trials). After each block, a pause was introduced, with a duration of at least 10 s and a maximum chosen by the participant. Instructions emphasized the inevitability of occasional errors and encouraged participants to strive for both speed and accuracy to minimize unnecessary waiting for the stop stimulus. Results obtained were analyzed using the “ANALYZE-IT” app provided with the “STOP-IT” implementation, with responses excluded automatically or manually according to the recommendations of Verbruggen et al.^69^,particularly if more than 20% of “go” trials were omitted.

During each trial, participants were presented with a fixation dot for 100 ms, followed by a white arrow pointing left or right. In “go” trials (75% of all trials) participants were instructed to press the “F” or “J” keys with their index finger depending on the arrow’s direction (“F” for the left arrow, “J” for the right arrow). In “stop” trials (25% of all trials), after a short delay (stop-signal delay; SSD), the arrow changed its color to red, signalling participants to suppress their response. The inter-trial interval was 400 ms, and the stimulus was presented for 100 ms. Initial SSD duration was 300 ms, which was increased by 50 ms if the participant successfully inhibited their response in the preceding trial or decreased by 50 ms if they failed to do so. The minimum value of SSD was 50 ms, with a fixed maximum reaction time of 1200 ms and an inter-trial interval of 400 ms.

#### Questionnaires

The NAS-50^60^ questionnaire measures self-control as a personality trait. This scale comprises 50 items categorized into five subscales: Initiative and Persistence (IP), Proactive Control (PC), Switching and Flexibility (SF), Inhibition and Adjournment (IA) and Goal Maintenance (GM). The NAS-50 demonstrates satisfying internal consistency, with a Cronbach’s alpha of 0.86 for the entire scale and ranging between 0.72 and 0.86 for subscales^60^. Responses are provided on a five-point Likert scale. For hypothesis testing, only the Inhibition and Adjournment (IA) subscale was utilized. The present internal consistency was α = 0.84.

The Barrat Impulsivity Scale-Brief (BIS-Brief)^62^ is a shortened version of the 30-item self-reported measure BIS-11^70^. It is the most commonly used questionnaire dedicated to the assessment of impulsivity, also in the field of GD^25^. Steinberg and colleagues^62^ demonstrated that BIS-Brief has similar construct validity which is observed for BIS-11, with Cronbach’s alpha of 0.78. Responses are recorded on a four-point Likert scale. In the current study, the Polish version of the BIS-Brief validated by Kata & Poleszak^72^ was utilized, showing an internal consistency of α = 0.69 (comparable to 0.73 obtained during the validation).

The Polish version of the Gaming Disorder Test^57,73^ was employed to assess GD risk. This scale comprises four items rated on a Likert scale. While the reliability obtained in our study (α = 0.73) was slightly lower than in the original (α = 0.84^57^), it remained acceptable.

#### Additional questionnaires for exploratory reasons

Direct gaming involvement (DGI) quantified as gaming time, was measured with the Gaming Involvement Scale^40^, which comprises six questions regarding the average time devoted to gaming per weekday and weekend day, along with engagement in various game-related activities such as watching game-related videos. The DGI score was derived by summing the values of two items from the Direct Gaming Involvement questions, reflecting gaming time exclusively.

The Barkley Deficits in Executive Function Scale-Short Form (BDEFS-SF)^59^ was utilized to assess deficits in daily activities. This 20-item short form assesses the frequency of engagement in specific types of executive functioning over the past six months, encompassing domains such as time management, self-organization/problem-solving, self-restraint, self-motivation, and self-regulation of emotion. The BDEFS-SF demonstrates high internal consistency, with a Cronbach’s alpha of 0.92 for the overall scale and ranging from 0.55 to 0.80 for subscales^59^. Items are based on a four-point Likert scale. In the current study, the internal consistency was α = 0.87 (from 0.73 to 0.88 for subscales).

The Self-Control Scale^64^, a 10-item questionnaire, was used to measure healthy impulse control. The measurement tool is based on a five-point Likert scale. The scale exhibits high internal consistency, with a Cronbach’s alpha of 0.89^64^. The Polish version of the questionnaire, validated by Pilarska & Baumeister^74^, was used in this study, demonstrating an internal consistency of α = 0.83.

The Emotion Regulation Questionnaire (ERQ)^61^ was used to assess two emotion regulation strategies: cognitive reappraisal and expressive suppression. This 10-item measure utilizes a seven-point Likert scale. The ERQ demonstrates satisfactory internal consistency, with alpha reliabilities averaging 0.79 for Reappraisal and 0.73 for Suppression^61^. The Polish version of the ERQ, validated by Szczygieł^75^, exhibited an internal consistency of α = 0.76 (α = 0.83 for cognitive reappraisal and α = 0.76 for expressive suppression) in the present study.

The BIS-BAS Scales^64^ were used to assess behavioral inhibition and activation. It is a four-factor questionnaire consisting of: (1) Behavioral Inhibition System (BIS; α = 0.74), (2) Behavioral Activation System (BAS) Reward Responsiveness (α = 0.73), (3) BAS Drive (α = 0.76), and (4) BAS Fun Seeking (α = 0.71). It comprises 20 items rated on a five-point Likert scale. The Polish version of the BIS-BAS Scales, validated by Müller & Wytykowska^76^, was utilized in this study. The internal consistency in this study was as the following: (1) BIS: α = 0.67, (2) BAS Reward Responsiveness: α = 0.47, (3) BAS Drive: α = 0.76, and (4) BAS Fun Seeking: α = 0.63.

Additionally, participants completed three questionnaires regarding their mental health, namely: Adult ADHD Self-Report Scale (ASRS-v1.1)^65^; Polish version^77^ Patient Health Questionnaire–4 (PHQ-4)^66^; Polish version^78^ and four items from IGD-20 Test^58^; Polish version^79^ to measure peripheral criteria of Gaming Disorder, namely Salience, Mood Modification, Tolerance, Withdrawal Symptoms. These measures were not included in the analyses.

### Statistical analyses

The primary focus of the analyses was to investigate the relationships among DGI, impulsivity, and the risk of GD.

To assess Hypotheses 1 and 2, Pearson correlation coefficients were computed to examine the strength and direction of the relationships among direct gaming involvement, impulsivity, and the risk of GD. The effect sizes of these correlations were assessed using Cohen’s guidelines.

Moving forward to Hypothesis 3, multiple linear regression analyses were conducted. Separate regression models predicting the risk of GD were constructed for each tool measuring impulsivity. Direct gaming involvement was introduced as a predictor in Model 1 (Step 1), and impulsivity was subsequently added in Model 2 (Step 2).

To test Hypothesis 4, a moderated regression analysis was conducted to investigate the potential interaction between direct gaming involvement and impulsivity in relation to GD risk. This analysis aimed to determine whether the relationship between DGI and GD risk varies depending on the level of impulsivity. Similar to Hypothesis 3, separate moderation analyses were conducted with each impulsivity measure as a moderator.

### Open science practices

Following established standards, the data, code and materials, along with the time-stamped pre-registration outlining the study protocol^81^, are available for download on the Open Science Framework. We pre-registered four hypotheses and the direction of additional exploratory analyses.

## Results

Statistical analyses were performed using IBM SPSS Statistics 29 software and PROCESS 4.1 macro. Descriptive statistics of all quantitative variables were presented in Supplementary Materials Table 1.

### Pre-registered hypotheses testing

We initiated the analysis by examining Pearson’s correlations among all variables specified in the pre-registration, assessing their associations with GD risk, DGI, and IGI. The correlation coefficients are detailed in Table 3.

**Table 3:** Pearson’s correlation matrix of impulsivity variables with GD, DGI, and IGI.

Variable	GD	DGI	IGI
GD	-	0.24*	0.20
DGI	0.24*	-	0.60***
IGI	0.20	0.60***	-
SSRT	− 0.24*	− 0.15	− 0.18
SST Go Error	− 0.01	− 0.13	− 0.06
GNG Go RT	− 0.01	− 0.10	− 0.13
GNG Stop Accuracy	− 0.14	0.09	− 0.03
GNG Go Accuracy	− 0.03	0.17	0.04
NAS50 Inhibition and Adjournment	− 0.26*	− 0.21	− 0.03
BIS-brief general	0.28*	0.20	0.18
BIS-brief Impulsivity in action	0.17	0.12	0.11
BIS-brief Self-control^a^	0.33**	0.24*	0.20

*** *p* < .001; ** *p* < .01; * *p* < .05.

^a^ Higher score indicates lower self-control.

Firstly, we examined the correlation between GD risk and gaming time (DGI), in order to verify Hypothesis 1. As expected, the correlation was significant, yet weak (*r* = 0.24; *p* < .01).

Subsequently, to assess Hypothesis 2, we evaluated the correlations between GD risk and various indicators of impulsivity. These included: two selected Stop-Signal Task results (Stop Signal Reaction Time (SSRT) and the proportion of incorrect responses on go trials with a response)^82^, selected measures from the Go/No-Go task (mean reaction time (GNG Go RT), proportion of correct responses (GNG Go Accuracy) and proportion of inhibited responses (GNG Stop Accuracy)^83^, Inhibition and Adjournment subscale from NAS-50^60^, as well as Barratt Impulsiveness Scale-Brief (BIS-Brief)^62^.

Moving on to Hypothesis 3, we addressed potential outliers by winsorizing one outlier within the dependent variable, adjusting GD score from 18 to 16. In the initial model construction, we identified heteroscedasticity as assessed by Breusch-Pagan test. The dependent variable was transformed using log base 10 transformation. This did not reduce heteroscedasticity, so we also transformed DGI due to its high skewness. Heteroscedasticity was no longer present in the models and the Breusch-Pagan test results rendered non-significant for all models.

Hierarchical regression models were then constructed in a stepwise manner, incorporating DGI in Step 1 and another independent variable in Step 2 (Table 4). Consistent with the regression assumptions, only variables significantly correlated with DGI were analysed.

**Table 4:** Results of regression analyses predicting GD risk based on DGI and impulsivity.

Model	Predictor	B	SE	Beta	t	*p*	F	ΔF	*R*²adj.	ΔR²
1	Constant	0.72	0.08		9.46	< 0.001	3.34		0.03	
	DGI_LOG	0.05	0.03	0.21	1.83	0.072				
2	Constant	0.91	0.13		6.80	< 0.001	3.18^*^	2.93	0.06	0.03
	DGI_LOG	0.04	0.03	0.17	1.47	0.146				
	SS_RT	0.00	0.00	− 0.20	-1.71	0.091				
1	Constant	0.76	0.07		10.40	< 0.001	1.95		0.01	
	DGI_LOG	0.04	0.03	0.15	1.40	0.166				
2	Constant	0.97	0.11		8.78	< 0.001	4.02^*^	5.97^*^	0.07	0.07
	DGI_LOG	0.03	0.03	0.13	1.12	0.229				
	NAS50 Inhibition and Adjournment	− 0.006	0.003	− 0.26	-2.44	0.017				
1	Constant	0.76	0.07		10.40	< 0.001	1.95		0.01	
	DGI_LOG	0.04	0.03	0.15	1.40	0.166				
2	Constant	0.65	0.09		7.39	< 0.001	3.73^*^	5.40^*^	0.06	0.06
	DGI_LOG	0.03	0.03	0.11	1.05	0.295				
	BIS-brief_general	0.01	0.00	0.25	2.32	0.023				
1	Constant	0.76	0.07		10.40	< 0.001	1.95		0.01	
	DGI_LOG	0.04	0.03	0.15	1.40	0.166				
2	Constant	0.64	0.08		7.67	< 0.001	4.47^*^	6.84^*^	0.08	0.07
	DGI_LOG	0.02	0.03	0.10	0.93	0.355				
	BIS-brief_Self_Control	0.02	0.01	0.28	2.62	0.011				

Dependent variable: GDT_LOG.

We started with building a model predicting GD risk based on DGI and SSRT. Due to 8 cases of missing SS_RT data, the analysis was performed on data from 74 participants. Initially, DGI was not a significant predictor of GD risk (*R*^2^ = 0.044, *F*(1, 72) = 3.338, *p* = 0.072; adjusted *R*^2^ = 0.031). However, adding SSRT in Step 2 resulted in the model being significant (*R*^2^ increase of 0.038, *F*(2, 71) = 2.928, *p* = 0.091), but the F-change was not significant.

Next, we built a model with DGI and NAS-50 Inhibition and Adjournment subscale as predictors. The initial model based on DGI was not significant (*R*^2^ = 0.024, *F*(1, 80) = 1.952, *p* = .166; adjusted *R*^2^ = 0.012), but the addition of Inhibition and Adjournment increased the explained variance to 7% and improved significance (*R*^2^ increase of 0.069, *F*(1, 79) = 5.970, *p* = 0.017).

Following this, we built a model with DGI and BIS-Brief general results. Similarly, the initial model results mirrored the previous outcomes. Yet, the addition of BIS-Brief increased the explained variance to 6% and improved the significance of the model (*R*^2^ increase of 0.062, *F*(1, 79) = 5.395, *p* = .023).

Finally, we performed the regression using only the Self Control subscale of BIS-Brief. We decided to do that since only this subscale was correlated with GD risk. Incorporating solely the Self Control subscale increased the statistical significance of the model and improved the explained variance to 8% (*R*^2^ increase of 0.078, *F*(1, 79) = 6.844, *p* = .011).

In order to test interactional Hypothesis 4, we performed a series of interaction analyses using each measure of impulsiveness as a moderator of the relationship between DGI and GD risk (both logarithmized). The results are presented in Table 5. In other words, we conducted separate analyses for general BIS-Brief and its Self-Control subscale, Inhibition and Adjournment subscale from NAS-50 and Stop-Signal (SSRT, SST Go Error) as well as Go/No-Go (GNG Go RT, GNG Stop Accuracy and GNG Go Accuracy) metrics as moderators.

**Table 5:** Results of moderation analyses testing interaction effects between GD, DGI, and impulsivity.

Effect	B	SE	t	*p*	LLCI	ULCI
Constant	0.23	0.50	0.46	0.644	− 0.77	1.23
DGI_LOG	0.29	0.18	1.61	0.112	− 0.07	0.65
SSRT	0.00	0.00	1.03	0.308	− 0.0021	0.0065
DGI_LOG * SSRT	− 0.00	0.00	-1.40	0.165	− 0.003	0.001
Constant	0.74	0.12	6.24	< 0.001	0.50	0.97
DGI_LOG	0.04	0.04	0.99	0.326	− 0.04	0.13
SST Go Error	-1.86	7.43	− 0.25	0.803	-16.68	12.96
DGI_LOG * SST Go Error	0.93	2.69	0.34	0.732	-4.43	6.28
Constant	-2.48	1.03	-2.40	0.019	-4.54	− 0.42
DGI_LOG	1.23	0.38	3.29	0.002	0.49	1.98
GNG Go RT	8.12	2.57	3.15	0.002	2.99	13.24
DGI_LOG * GNG Go RT	-3.00	0.94	-3.20	0.002	-4.87	-1.13
Constant	0.66	0.64	1.04	0.303	− 0.61	1.94
DGI_LOG	0.15	0.27	0.56	0.577	− 0.38	0.68
GNG Stop accuracy	0.00	0.01	0.15	0.881	− 0.01	0.02
DGI_LOG * GNG Stop accuracy	− 0.00	0.00	− 0.42	0.677	− 0.01	0.01
Constant	3.79	3.68	1.03	0.306	-3.54	11.11
DGI_LOG	-1.25	1.59	− 0.78	0.437	-4.42	1.93
GNG Go Accuracy	− 0.03	0.04	− 0.82	0.414	− 0.10	0.04
DGI_LOG * GNG Go Accuracy	0.01	0.02	0.81	0.423	− 0.02	0.05
Constant	1.05	0.44	2.36	0.021	0.16	1.93
DGI	0.00	0.16	0.01	0.991	− 0.32	0.32
NAS50 Inhibition and Adjournment	− 0.01	0.01	− 0.61	0.545	− 0.04	0.02
DGI * NAS50 Inhibition and Adjournment	0.00	0.00	0.19	0.852	− 0.01	0.01
Constant	0.80	0.24	3.32	0.001	0.32	1.28
DGI	− 0.04	0.09	− 0.39	0.700	− 0.22	0.15
BIS Self Control	− 0.00	0.03	− 0.01	0.993	− 0.05	0.05
DGI * BIS Self Control	0.01	0.01	0.68	0.497	− 0.01	0.03
Constant	0.91	0.27	3.37	0.001	0.37	1.45
DGI	− 0.08	0.10	− 0.74	0.461	− 0.28	0.13
BIS_general	− 0.01	0.02	− 0.48	0.632	− 0.04	0.02
DGI * BIS_general	0.01	0.01	1.05	0.299	− 0.01	0.02

Only the interaction between Go/No-Go Reaction Time and Direct Gaming Involvement turned out to be significant, aligning with Hypothesis 4 (*b* = -3.001, *β* = − 0.4295 *SE* = 0.9393 *p* = 0.002, *R*^2^ increase = 0.1157). The effect was significant for participants with GNG Go RT below 0.392 ms (70 percentile) and above 0.444 ms (96,25 percentile). This means that the interaction effect was not significant only among 26,25% of participants with GNG Go reaction time above 0.392 and below 0.444. The remaining interactions between DGI and impulsiveness measures were statistically non-significant.

### Exploratory analyses

We decided to take the opportunity to examine potential relationships between GD risk and several variables not directly related to impulsiveness. Unlike the results reported above, they were not the subject of hypotheses, yet their exploration was indicated during pre-registration. Detailed results can be found in Table 6 for correlations and Table 7 for regression analyses.

**Table 6:** Pearson’s correlation matrix of exploratory variables with GD, DGI, and IGI.

Variable	GD	DGI	IGI
GD	-	0.24*	0.20
DGI	0.24*	-	0.60***
IGI	0.20	0.60***	-
SSD	0.03	0.08	0.11
SST Go RT	0.00	0.05	0.08
GNG Total Accuracy	− 0.12	0.13	− 0.02
Peripheral GD	0.20	0.05	0.21
BDEFS Self Management Time	0.36***	0.12	0.08
BDEFS Organization ProblemSolving	0.31**	− 0.04	0.07
BDEFS Self Restraint	0.14	0.10	0.09
BDEFS Self Motivation	0.42***	0.22*	0.25*
BDEFS Self Regulation Emotions	0.19	− 0.08	− 0.09
NAS50 Proactive Control	− 0.32**	− 0.20	− 0.20
NAS50 Goal Maintenance	− 0.18	− 0.23*	− 0.11
NAS50 Initiative and Persistence	− 0.39***	− 0.36***	− 0.25*
NAS50 Switching and Flexibility	− 0.12	0.12	0.02
ERQ Cognitive Reappraisal	− 0.03	0.25*	− 0.07
ERQ Expressive Suppression	0.05	0.16	0.24*
Emotional Regulation General	0.00	0.28*	0.09
Behavioral Activation Drive	− 0.16	0.02	0.02
Behavioral Activation Fun Seeking	0.14	0.08	0.12
Behavioral Activation Reward Responsiveness	− 0.04	0.05	− 0.04
Behavioral Inhibition	0.06	0.06	0.13
Self control (SCS-SF)	− 0.43***	− 0.29**	− 0.25*

*** *p* < .001; ** *p* < .01; * *p* < .05.

^a^ Higher score indicates lower self-control.

**Table 7:** Results of regression analyses predicting GD risk based on DGI and cognitive variables that were the subject of exploration.

Model	Predictor	B	SE	Beta	t	*p*	F	ΔF	*R*²adj.	ΔR²
1	Constant	0.76	0.07		10.40	< 0.001	1.95		0.01	
	DGI_LOG	0.04	0.03	0.15	1.40	0.166				
2	Constant	0.60	0.08		7.44	< 0.001	5.26^***^	6.24^***^	0.17	0.16
	DGI_LOG	0.02	0.03	0.09	0.91	0.366				
	BDEFS_Self_Management_Time	0.01	0.01	0.18	1.38	0.173				
	BDEFS_Organization_ProblemSolving	0.01	0.01	0.11	0.92	0.362				
	BDEFS_Self_Motivation	0.01	0.01	0.24	1.78	0.080				
1	Constant	0.76	0.07		10.40	< 0.001	1.95		0.01	
	DGI_LOG	0.04	0.03	0.15	1.40	0.166				
2	Constant	1.22	0.14		8.96	< 0.001	4.96^***^	5.84^**^	0.16	0.15
	DGI_LOG	0.00	0.03	< 0.01	0.07	0.946				
	NAS50 Proactive Control	0.00	0.00	− 0.17	-1.56	0.123				
	NAS50 Initiative and Persistence	− 0.01	0.002	− 0.28	-2.37	0.020				
	NAS50 Inhibition and Adjournment	− 0.003	0.003	− 0.13	-1.13	0.263				
1	Constant	0.76	0.07		10.40	< 0.001	1.95		0.01	
	DGI_LOG	0.04	0.03	0.15	1.40	0.166				
2	Constant	1.08	0.10		1.62	< 0.001	9.68^***^	17.01^***^	0.18	0.16
	DGI_LOG	0.01	0.03	0.05	0.53	0.598				
	Self_control	− 0.01	0.00	− 0.43	-4.12	< 0.001				

Note Dependent variable: GDT_LOG.

#### Lack of association between demographic characteristics and GD risk

There was no correlation between GD risk and age of the participants (*r* = 0.02, *p* = 0.894). Additionally, we found no difference between GD risk between participants divided by gender (95% CI, -1.82 to 0.74), *t*(77) = 0.844, *p* = 0.401), marital status (95% CI, -0.77 to 1.52), *t*(80) = 0.649, *p* = 0.518) and employment status (95% CI, -1.19 to 1.11), *t*(80) = − 0.073, *p* = 0.942).

#### Correlations between GD risk and deficits in executive functioning

Correlation analyses between GD risk and deficits in executive functioning (individual subscales of Barkley Deficits in Executive Functioning Scale^84^ showed that GD may be related to several categories of deficits in executive functioning. Among the five areas examined, were found to be correlated: Time Management (*r* = 0.36; *p* < 0.001), Organization and Problem Solving (*r* = 0.31; *p* < 0.01) and Self-Motivation (*r* = 0.42; *p* < .001).

#### Correlations between GD risk and self-control

Another interesting area of cognitive abilities was explored through scales for self-control assessment (NAS-50)^60^. In addition to the Inhibition and Adjournment subscale, which was the subject of the previously tested hypotheses, two other subscales, specifically Proactive Control (*r* = − 0.32; *p* < 0.01) and Initiative and Persistence (*r* = − 0.39; *p* < 0.001), also negatively correlated with GD risk.

Furthermore, results of another measure of self-control, the Self-Control Scale Short Form^65^, were also negatively correlated with GD risk (*r* = − 0.43; *p* < 0.001).

#### No correlations between behavioral inhibition/activation and emotional regulation and GD risk

On the other hand, we found no correlation between GD risk and behavioral inhibition system or behavioral activation system measured with BIS/BAS Scales. Similarly, no correlations were found between GD risk and emotional regulation processes (expressive suppression and cognitive reappraisal).

#### Self-control and executive functioning as potential predictors of GD risk

Based on the linear correlations described above, we further explored the potential of incorporating self-control and deficits in executive functioning variables to enhance the predictive accuracy of the regression model estimating GD risk based solely on DGI. We found that including the scores of all three subscales examining deficits in cognitive functioning to the equation significantly improved the percentage of explained variance in the model by 16% (*R*^2^ increase of 0.16, *F*(4, 77) = 5.261, *p* < 0.001). However, none of the individual predictors emerged as statistically significant.

The addition of variables related to self-regulation resulted in a similar way. When scores from three subscales of NAS50 (which were found to correlate with GD risk) were taken into account in the regression, the percent of the explained variance increased by 15% (*R*^2^ increase of 0.15, *F*(4, 77) = 4.960, *p* < 0.001). Notably, only the Initiative and Persistence subscale turned out to be a statistically significant predictor of GD risk. Moreover, the inclusion of Self-Control Scale results in the regression further increased the percent of explained variance (*R*^2^ increase of 0.16, *F*(2, 79) = 9.677, *p* < 0.001), with self-control emerging as a significant predictor of GD risk.

## Discussion

This study employs a dual approach to the operationalization of impulsivity, aiming to comprehensively explore its relationship with GD. While the link between gaming and cognitive traits has long been investigated, initially focusing on its impact on cognitive skill development, there’s a growing recognition of its multifaceted nature. There is a gradual increase in interest in research on the role of cognitive variables in GD development. One of the variables that emerge in this context is self-regulation. Within it, we focused on impulsivity. Our study sought to validate prior findings^25^, while also examining the hypothesis regarding impulsivity’s moderating role in the DGI-GD risk relationship. To our knowledge, this hypothesis was tested for the first time.

We confirmed hypotheses concerning the relationship between impulsivity and GD risk, while rejecting the hypothesis of impulsivity’s moderating role. However, the most intriguing insights stem from our exploratory analyses, suggesting that among the variables related to self-regulation, characteristics related to the reflective system may play a leading role in the context of GD development. In the following discussion, we contextualize these findings within the Tripartite Neurocognitive Model of Internet Gaming Disorder^85^.

### Pre-registered hypotheses testing

Overall, our findings confirm the previous empirical data concerning the connections between GD risk and gaming involvement (Hypothesis 1) as well as impulsivity (Hypothesis 2), particularly evident in self-report data. All expected correlations between self-reported impulsiveness and GD risk, excluding a single subscale (Impulsivity in action from BIS-Brief), have been obtained. On the other hand, only one out of five behavioral metrics of impulsiveness (average time to stop a response in a Stop Signal Task; SS_RT) correlated with GD risk. Therefore, while Hypothesis 2 cannot be fully confirmed, it finds partial validation in self-report data.

Furthermore, self-report measures of impulsivity underscored their significance in regression models predicting GD risk levels based on the DGI (Hypothesis 3). As expected, adding a variable reflecting impulsivity to the equation improved the model fit in a statistically significant way each time. However, incorporating the only behavioral metric of impulsivity showing a linear relationship with GD risk (SS_RT) into the regression equation failed to align with our predictions. Once again, a clear difference between self-report and objective measures emerged.

Hypothesis 4, postulating the interaction between DGI and impulsiveness, was not supported. While one regression equation indicated statistical significance for this interaction (DGI*GNG Go RT), given the numerous moderators tested, this finding alone does not sufficiently confirm the interaction between DGI and impulsivity. In other words, it should be recognized that, in the light of the data we have collected, individual characteristics regarding impulsivity do not influence the strength of the relationship between time spent gaming and the risk of GD. Nevertheless, there is a possibility that future research examining the relationships between DGI and the reaction time in Go/No Go task will confirm our results.

In summary, DGI and impulsivity can be considered complementary yet independent predictors of GD risk.

#### The difference between the results of self-reported and behavioral indicators of impulsiveness

The inconsistency between the results obtained using self-report and objective variables is intriguing. Firstly, our dataset clearly exhibits this difference. Secondly, such results may seem contradictory to the previously obtained results utilizing computerized measures of impulsivity. In a recent review of 33 studies exploring the relationship between impulsivity and GD, all but one study clearly demonstrated a correlation between these variables^25^. Our findings confirm this relationship in the case of self-report data, while almost entirely contradicting it in the case of behavioral data. However, it is worth noting that the pattern of correlation between computer-measured impulsivity and GD found in previous studies is far less obvious than it might seem. Only one study identified a correlation between impulsivity (operationalized through a Single Key Impulsivity Task) and GD^86^. In most cases, studies using objective measures of impulsivity utilized experimental design and correlations were entirely absent from the reported findings^26,87–92^. In one study, researchers reported a lack of statistically significant correlations^93^. Hence, while the relationship between GD and impulsivity measured through self-report tools is empirically established, there is currently too little evidence to conclude a correlation between impulsivity measured via computer tasks and GD. Therefore, our findings should not be viewed as contradictory to the current consensus. Nonetheless, this lack of relationship is puzzling considering that both computer-based and self-report tasks should refer to the same variable, impulsivity.

One potential explanation for this inconsistency in results across different measurement methods is the potentially inadequate selection of computer tasks. Prior studies predominantly utilized the Stop Signal Task and Go/No Go Task, also employed in our research. It is conceivable that these tasks capture aspects of impulsivity that are simply unrelated to GD. In contrast, the Single Key Impulsivity Task, the results of which correlated with GD^86^, captures tolerance for delayed rewards rather than the ability to respond or withhold responses to simple stimuli.

Another plausible explanation lies in the observations of Brevers and colleagues^94^, who noted that although computer tasks provide the opportunity to accurately measure behavioral parameters, this goes hand in hand with their low ecological validity^94^. Firstly, the results of computer tasks may not consistently align with real-life challenge performance^95^. What may be even more important for the research on gamers is the fact that since the control of computer-based tasks is similar to playing video games, it is possible to transfer computer proficiency gained for gaming purposes to measurement tasks. For instance, poorer inhibition results achieved by gamers during hybrid Go/ No-Go and Stop Signal tasks^96^ may indicate adaptation to gaming conditions rather than increased impulsivity itself^94^. Consequently, if the factors influencing the results of such tasks overlap (impulsivity as a trait and environmental adaptation), the interpretation of the findings becomes blurred and limited. Thus, our results, alongside those previously reported, do not cast doubt on the connection between impulsivity and GD. However, it suggests that more refined computer tasks or self-report measures may be necessary in such cases.

#### No interaction between DGI and impulsivity on GD risk

As demonstrated by Hu et al.^29^, impulsivity can act as a moderator in the relationship between positive affective associations with gaming and GD. In individuals with higher levels of impulsivity, the positive relationship between positive affect towards gaming and GD was stronger compared to their less impulsive counterparts. This finding aligns with models of addiction emphasizing the importance of joint, interactive influence of many factors in addiction development. Both the Interactional-Transformational Model, which emphasizes the importance of the interaction of various factors in the development of pathology^53^, and the I-PACE model^9^ could serve here. While Hu and colleagues^29^ did not gather data on gaming involvement, it can be assumed that, in principle, positive affect towards gaming could be strongly related to the time devoted to gaming. Therefore, we expected that employing a similar analytical approach would reveal a comparable interaction between DGI and impulsivity for GD.

However, our predictions were not confirmed. Regardless of how impulsivity was operationalized, no statistically significant interaction with DGI was observed. Nonetheless, it should be noted that our sample differed in age, gender proportion and size compared to the study by Hu et al. Additionally, Hu and colleagues^29^ used a slightly different operationalization of impulsivity and GD, conducting analyses on impulsivity after dichotomization and excluding cases falling within ∓1 standard deviation. Such analyses would not be viable in our case due to the limited sample size. Hence, premature dismissal of further investigation based solely on these findings would be unwarranted. Further research is certainly necessary, including an attempt at a modified replication of the study by Hu et al.^29^. This replication should aim to uphold the integrity of the original research while introducing a singular modification: substituting the evaluation of positive affect towards gaming with an assessment of gaming involvement.

#### Results of exploratory analysis in the context of the tripartite neurocognitive model of internet gaming disorder

Recently, Wei and colleagues summarized research findings related to the neurocognitive processes underlying GD^85^. As a result, they proposed the triadic system that governs behavior and decision-making associated with addictive disorders, including gaming disorder^86^. This tripartite system includes three distinct subsystems: (1) the impulsive system, which often mediates fast, automatic, unconscious, and habitual behaviors; (2) the reflective system, which mediates deliberating, planning, predicting future outcomes of selected behaviors, and exerting inhibitory control; and (3) the interoceptive awareness system, which generates a state of craving through the translation of somatic signals into a subjective state of drive. The authors postulate that GD formation may be associated with (1) a hyperactive “impulsive” system, (2) a hypoactive “reflective” system, as moderated by (3) an interoceptive awareness system.

While our study was not directly inspired by this model, we planned exploratory analyses on variables that align with these three systems to some extent. In attempting to speculate how to categorize the tools we employed into each system, we cautiously propose, with some degree of uncertainty, measures that offer insights into each subsystem. Our retrospective proposal for tool allocation to these systems can be found in Table 8.

**Table 8:** Proposal for assigning individual variables used in the study to systems in the tripartite model.

Variable	System		
	Impulsive	Reflective	Interoceptive
SSD	?	?	
SSRT	?	?	
SST Go RT	?	?	
SST Go Error	?	?	
GNG Go RT	?	?	
GNG Stop Accuracy	?	?	
GNG Go Accuracy	?	?	
GNG Total Accuracy	?	?	
BDEFS Self Management Time		+	
BDEFS Organization ProblemSolving		+	
BDEFS Self Restraint		+	
BDEFS Self Motivation		+	
BDEFS Self Regulation Emotions			+
NAS50 Proactive Control		+	
NAS50 Goal Maintenance		+	
NAS50 Initiative and Persistence		+	
NAS50 Inhibition and Adjournment	+		
NAS50 Switching and Flexibility		?	
ERQ Cognitive Reappraisal			+
ERQ Expressive Suppression			+
Emotional Regulation General			+
Behavioral Activation Drive	+		
Behavioral Activation Fun Seeking	+		
Behavioral Activation Reward Responsiveness	+		
Behavioral Inhibition			?
Self control (SCS-SF)		+	
BIS-Brief Impulsivity in action	+		
BIS-Brief Self-control		+	

+ the measure can be treated as a metric of this system; ? the measure can probably be treated as a metric of this system.

It is important to acknowledge the uncertainty surrounding computer-based measures, as while most researchers typically consider them indicators of impulsivity, there is an intuitive inclination to assign them to the impulsive system. However, the authors of the Tripartite Model directly attribute these measures to the reflective system^85^. Regarding the Switching and Flexibility subscale of NAS-50^61^, while the content of individual items may imply a connection to the Reflective System, it’s crucial to note that Wei’s Model^85^ refers directly to the development of behavioral addictions. The items in this subscale clearly refer to tasks requiring multitasking or the speed of learning new action patterns^97^. Finally, considering the Behavioral Inhibition subscale of the BIS/BAS^63,76^, based on the content of individual items, it should be treated as a metric of the Interoceptive System despite the name suggesting affiliation with the Reflective System.

Upon comparing the results of our exploratory analyzes with our proposed classification of individual measures into individual systems, an interesting regularity can be noticed. Measures corresponding to the Impulsive rarely correlate with GD risk; specifically, Inhibition and Adjournment (NAS-50) and no one related to Interoceptive System correlated. In contrast, almost all measures assigned to the Reflective System correlate with GD risk, with only the Self-Restraint (BDEFS) and Goal Maintenance (NAS-50) subscales showing no correlation. While this exploratory analysis based on a post-factum classification cannot be considered conclusive, it underscores the need for future research to rigorously test the predictions of Wei’s model. This necessitates a more structured approach, including possible primary role of the Reflective System compared to the other two. To do this, it will be necessary to carefully analyze the theoretical postulates of the authors of the model and select appropriate operationalizations, also considering tools that we did not use in the reported study.

## Limitations and further research directions

The reported study had limitations that must be taken into account when assessing its significance for understanding the relationship between impulsivity and GD risk. Firstly, our sample, although calculated with the planned analyses in mind, was relatively small, which made it impossible to conduct more advanced exploratory analyses. Moreover, it was a convenience sampling, only taking into account the key eligibility criteria. On the other hand, it was diverse in terms of gender and age. It is also important to note that the study focused on a specific demographic—students from one university—which may limit the generalizability of the findings to other populations. Differences in gaming habits and impulsivity across age groups, socioeconomic statuses, and geographic regions mean that the results may not fully apply to broader populations. Therefore, caution is warranted when extending our conclusions beyond the study’s context, especially in terms of gaming disorder (GD) risk. Future research should aim to collect data from a more diverse population of gamers, which would allow for more representative results and broader applicability.

The observed inconsistencies between self-reported and behavioral measures of impulsivity suggest that these measures may reflect different aspects of impulsivity. Given that self-reported impulsivity was more predictive of GD risk than behavioral tasks, we must exercise caution in making broad claims about the relationship between impulsivity and GD risk. While self-reported data may highlight impulsivity traits more aligned with gaming behavior, further research is required to validate and expand upon these findings, ideally through the use of more targeted or refined measurement tools.

In the future studies on the relationship between impulsivity and GD, it will be necessary to take into consideration several improvements such as longitudinal study design with larger, more diverse samples and improved measurement techniques. The advantage of our study was the fact that it was pre-registered and we used precisely selected and previously validated measurement tools. However, the multitude of tools utilized for exploratory analyses posed a risk of inducing participant fatigue. In future research, the standards of tool quality and pre-registration protocols should be maintained, yet it would certainly be beneficial to reduce the number of tools used. It should be done on a basis of theoretical premises and the empirical data collected and presented in this paper. This would be a particularly challenging task when attempting to further empirically verify the significance of the individual systems identified within the Tripartite Model^85^. Given the study’s cross-sectional design, drawing definitive conclusions about cause-and-effect relationships is constrained. Subsequent studies should examine the relationships longitudinally.

## Conclusions

In this study, we successfully validated previous findings concerning the relationship between gaming time, impulsiveness, and GD risk. Additionally, we discovered that these predictors can be viewed as complementary factors. Nonetheless, contrary to our expectations, we did not confirm the interaction between these variables affecting GD risk. Although a definitive rejection of this prediction may require the collection of additional data, it should be considered a first indication that there is no interplay between gaming time and impulsivity in the context of the development of GD. These findings should be interpreted with a degree of caution. The limitations of our study—such as sample specificity and measurement challenges—suggest that further research is essential before drawing broader conclusions. The exploratory analyzes conducted provided compelling data that align with the predictions of the Tripartite Model of Internet Gaming Disorder^85^. However, it’s crucial to note that our study was not specifically designed to test this model. Thus, there remains a critical need for further research in this direction.

## Data Availability

The datasets generated during and/or analysed during the current study are available in the OSF repository, https://doi.org/10.17605/OSF.IO/5E8UG.
